# Effect of Chronic Kidney Diseases on Mortality among Digoxin Users Treated for Non-Valvular Atrial Fibrillation: A Nationwide Register-Based Retrospective Cohort Study

**DOI:** 10.1371/journal.pone.0160337

**Published:** 2016-07-28

**Authors:** Maurizio Sessa, Annamaria Mascolo, Mikkel Porsborg Andersen, Giuseppe Rosano, Francesco Rossi, Annalisa Capuano, Christian Torp-Pedersen

**Affiliations:** 1 Department of Experimental Medicine, Section of Pharmacology L. Donatelli, Second University of Naples, Naples, Italy; 2 Department of Health, Science and Technology, Aalborg University, Aalborg, Denmark; 3 IRCCS San Raffaele Pisana, Rome, Italy; 4 Cardiovascular and Cell Sciences Research Institute, St. George's University of London, London, United Kingdom; Medizinische Universitat Innsbruck, AUSTRIA

## Abstract

**Purpose:**

This study investigated the impact of chronic kidney disease on all-causes and cardiovascular mortality in patients with atrial fibrillation treated with digoxin.

**Methods:**

All patients with non-valvular atrial fibrillation and/or atrial flutter as hospitalization diagnosis from January 1, 1997 to December 31, 2012 were identified in Danish nationwide administrative registries. Cox proportional hazard model was used to compare the adjusted risk of all-causes and cardiovascular mortality among patients with and without chronic kidney disease and among patients with different chronic kidney disease stages within 180 days and 2 years from the first digoxin prescription.

**Results:**

We identified 37,981 patients receiving digoxin; 1884 patients had the diagnosis of chronic kidney disease. Cox regression analysis showed no statistically significant differences in all-causes (Hazard Ratio, HR 0.89; 95% confident interval, CI 0.78–1.03) and cardiovascular mortality (HR 0.88; 95%CI 0.74–1.05) among patients with and without chronic kidney disease within 180 days of follow-up period. No statistically significant differences was found using a 2 years follow-up period neither for all causes mortality (HR 0.90; 95%CI 0.79–1.03), nor for cardiovascular mortality (HR 0.87; 95%CI 0.74–1.02). No statistically significant differences was found comparing patients with and without estimated Glomerular Filtration Rate <30ml/min/1.73m^2^ and patients with different stages of chronic kidney disease, for all-causes and cardiovascular mortality within 180 days and 2 years from the first digoxin prescription.

**Conclusions:**

This study suggest no direct effect of chronic kidney disease and chronic kidney disease stages on all-causes and cardiovascular mortality within both 180 days and 2 years from the first digoxin prescription in patients treatment-naïve with digoxin for non-valvular atrial fibrillation.

## Introduction

A main strategy to treat atrial fibrillation is heart rate control. Heart rate control strategy includes the administration of one or more heart rate controlling agents, including non-dihydropyridine calcium channel antagonists, beta-blockers, and digoxin [1–4]. After the post-Digoxin Investigation Group trial era [5], digoxin is commonly used worldwide as heart rate control agent in the treatment of atrial fibrillation [6]. Studies have examined the effect of digoxin on mortality [7–28], but no single study have directly evaluated the effect of chronic kidney disease on mortality in patients that receive digoxin for atrial fibrillation. European Heart Rhythm Associations position paper for heart rate control therapy in patients with chronic kidney disease [29], endorsed by American and Asian Heart Rhythm Association, recommend the same treatment to patients with or without kidney disease with appropriate adjustment of dose according to glomerular filtration rate [29]. Atrial fibrillation and kidney disease are commonly found in the same patient and prior studies have shown that the presence of one condition increased the likelihood of finding the other [30–33]. Patients with chronic kidney disease have generally been excluded from clinical trials, therefore register based studies are currently the best opportunity to gain further insight [29,33].

The objective of this study was therefore to investigate the impact of chronic kidney disease on all-causes and cardiovascular mortality when patients with non-valvular atrial fibrillation initiate treatment with digoxin. The study included the entire Danish population between 1997 and 2012.

## Methods

### Data sources

All Danish residents are assigned a permanent Civil Personal Register number. This 10-digit number makes possible to link information from different registers to an individual patient [34]. Through this identifier, it was possible to link data on prescription fills, hospitalizations diagnosis, laboratory analysis, surgical procedures, the cause of death and vital status. The information was collected in the: Danish National Patient Registry [35], Danish Registry of Medicinal Product Statistics [36], Danish Civil Registration System [37], Clinical laboratory information system [38] and the National Causes of Death Registry [39]. The original role of each administrative registry is described elsewhere [35–39].

### Study population

All patients with non-valvular atrial fibrillation and/or atrial flutter from January 1, 1997 to December 31, 2012 were extracted. The diagnosis of atrial fibrillation have been validated in the Danish Patient Registry and found to have a verification rate of 99% among hospitalized patients [40].

From this preliminary population were extracted only digoxin treatment-naïve users who have initiated pharmacological treatment for non-valvular atrial fibrillation in the period from January 1, 1997. Patients were excluded if they started digoxin in co-administration with other antiarrhythmic drugs (including beta-blockers). Treatment initiation date of digoxin was used as the index date for each patient, as all patients should be diagnosed with non-valvular atrial fibrillation within the index date.

### Study covariates

Age, gender and vital status were obtained at index date. Major comorbidities were evaluated considering hospitalization diagnosis prior or equal to index date. Hospitalization diagnosis codes used are shown in S1 Table and was validated in previous studies [41,42]. Pharmacological treatment other than digoxin was obtained by claimed prescription up to 180 days before the index date; for this purpose, Anatomical Therapeutic Classification codes were used and the relative codes were defined in S2 Table. Evaluation of mean serum potassium and haemoglobin concentration were obtained based on laboratory sample results up to 180 days before the index date. The risk of stroke/thromboembolism for all patients was assessed at the index date according to the CHA2DS2-VASc score (C = Congestive heart failure; H = Hypertension; A = Age; D = Diabetes; S = Stroke; V = Vascular disease; sc = Sex category), as described elsewhere [43].

### Outcomes

The study outcomes were all-causes and cardiovascular mortality within 180 days and 2 years from the index date.

### Statistical analysis

Baseline characteristics were compared using the t-tests for continuous variables and χ^2^ for categorical variables. Log-rank test was assessed to compare survival curves. All analyses were based on intention-to-treat and used a statistically significant level of p<0.05 (2-sided). All the unadjusted analysis were performed using Cox regression analysis with only outcome and exposure.

In the main analysis, the study population was divided into two cohorts: patients with or without chronic kidney disease. We classified a patient as having chronic kidney disease if the patient had a diagnosis of one of the following pathologies classified as International Classification of Diseases v.10 codes: N02-08,11,12,14,15.8–16.0,16.2–16.4,16.8,18,19,26, Q61.2,61.3,61.5,61.9, E10.2,11.2,13.2,14.2 I12.0, M30.0,31.3,31.9,32.15. Patients should be diagnosed with chronic kidney disease before index date to be included. In the crude analysis, survival probability was estimated using Kaplan–Meier method for all-causes mortality and cumulative incidence for cardiovascular mortality. Cox proportional hazard model was used to compare the adjusted risk of all-causes and cardiovascular mortality between the two cohorts. Cox regression was adjusted for age, sex, year of inclusion, diagnosis of alcohol abuse, myocardial infarction, diabetes mellitus, heart failure, hypertension, cancer, chronic obstructive pulmonary disease (COPD), liver disease, syncope, peripheral arterial disease, ventricular arrhythmia, serum haemoglobin and potassium concentration, stroke or systemic thromboembolism history, CHA2DS2-VASc score, digoxin dosage and drugs listed in S2 Table. Subjects were followed up for 180 days and 2 years from index date and censored at death or at the end of the follow-up period on December 31, 2012. The assumptions of Cox models were tested, and the models were found valid. Five sensitivity analyses were performed. In the first sensitivity analysis, propensity score was used to match patients with chronic kidney disease (exposed) with four patients without chronic kidney disease (unexposed). This technique matched the exposed and unexposed by multivariate conditional logistic regression analysis on age, sex, year of inclusion, diagnosis of myocardial infarction, peripheral arterial disease, alcohol abuse, bleeding history, chronic obstructive pulmonary disease, diabetes mellitus, heart failure, hypertension, liver disease, stroke or systemic thromboembolism history, CHA2DS2-VASc score, digoxin dosage and drugs listed in S2 Table. Cox proportional hazard analysis was performed on the propensity-matched subpopulation to compare the adjusted risk of all-causes and cardiovascular mortality between the two cohorts.

In the second sensitivity analyses, the subpopulation of patients with concurrent creatinine measurement within 180 days before index date was selected from the study population. Applying chronic kidney disease epidemiology collaboration creatinine-based equation [44], we established the estimated Glomerular Filtration Rate (eGFR). Based on the results of eGFR, patients were divided into two cohorts, those with eGFR <30 and those with eGFR ≥ 30 ml/min/1.73m^2^. Each patient with eGFR <30 ml/min/1.73m^2^ was propensity matched with four patients with eGFR ≥ 30 ml/min/1.73m^2^ using the method previously described. Cox proportional hazard analysis was performed on the propensity-matched subpopulation to assess the adjusted risk of all-causes and cardiovascular mortality between the two cohorts. Survival curves were generated to compare cumulative mortality rate for both cohorts.

In the third sensitivity analysis, the subpopulation of patients that started digoxin therapy from 1 January 1997 to 31 December 2010 was selected. Considering that the end of follow-up period was 31 December 2012, this guarantee a potential follow-up period of at least 2 years for each patient. Three different analysis were performed on this subpopulation generating three different parts of the third sensitivity analysis. In the part one, we performed the main analysis using a two-year follow-up period. In parts two and three, we performed the first and second sensitivity analysis using a two-year follow-up period.

In the fourth sensitivity analysis, we performed the same methods as in the second sensitivity analysis and part three of third sensitivity analysis using eGFR as continuous predictor and multi-categorical predictor. In particular, when eGFR was used as multi-categorical predictor, five groups were generated according to the five stages defined into Kidney Disease Outcome Quality Initiative clinical practice guidelines [45]. In particular, the five stages considered were: Stage 5: eGFR < 15; Stage 4: 15≤ eGFR ≤29; Stage 3: 30≤ eGFR ≤59; Stage 2: 60≤ eGFR ≤89; Stage 1: eGFR ≥90; eGFR measurement unit: ml/min/1.73m^2^.

In the fifth sensitivity analysis, we performed the same methods as in the main analysis using a narrow definition of chronic kidney disease. A patient was classified as having a chronic kidney disease if he/she had a diagnosis of one of the following International Classification of Diseases v.10 codes: N18.3, N18.4, N18.5, N18.6 and N18.9.

Persistence of digoxin treatment was assessed within 180 days and 2 years follow-up period from index date. Persistence was defined as the proportion of patients in digoxin treatment on each relative day throughout each of the individual treatment periods starting at index date until patient death or at the last digoxin prescription date. This could be assessed based on the same assumptions of the calculations of the daily dosages, as described elsewhere [46]. An additional analysis was performed using persistence of digoxin treatment as time of exposure to digoxin to assess the risk of all-causes and cardiovascular mortality among patients with and without chronic kidney disease within 180 days and 2 years follow-up period. In particular, the same methods described in the main analysis and in the third sensitivity analysis—part one were used respectively, however using persistence of digoxin treatment as time of exposure to digoxin.

Data management was performed using SAS statistical software (version 9.4, SAS Institute Inc., Cary, North Carolina) and data analysis was performed using R (version 3.2.2, R Development Core Team). Nearest-neighbor propensity score matching was implemented by using the MatchIt package in the R statistical program.

### Compliance with ethical standards

In Denmark, register-based retrospective studies do not require ethical approval. The study was approved by the Danish Data Protection Agency. Patient records/information was anonymized and de-identified prior to analysis.

## Results

### Main analysis

**Fig 1:**
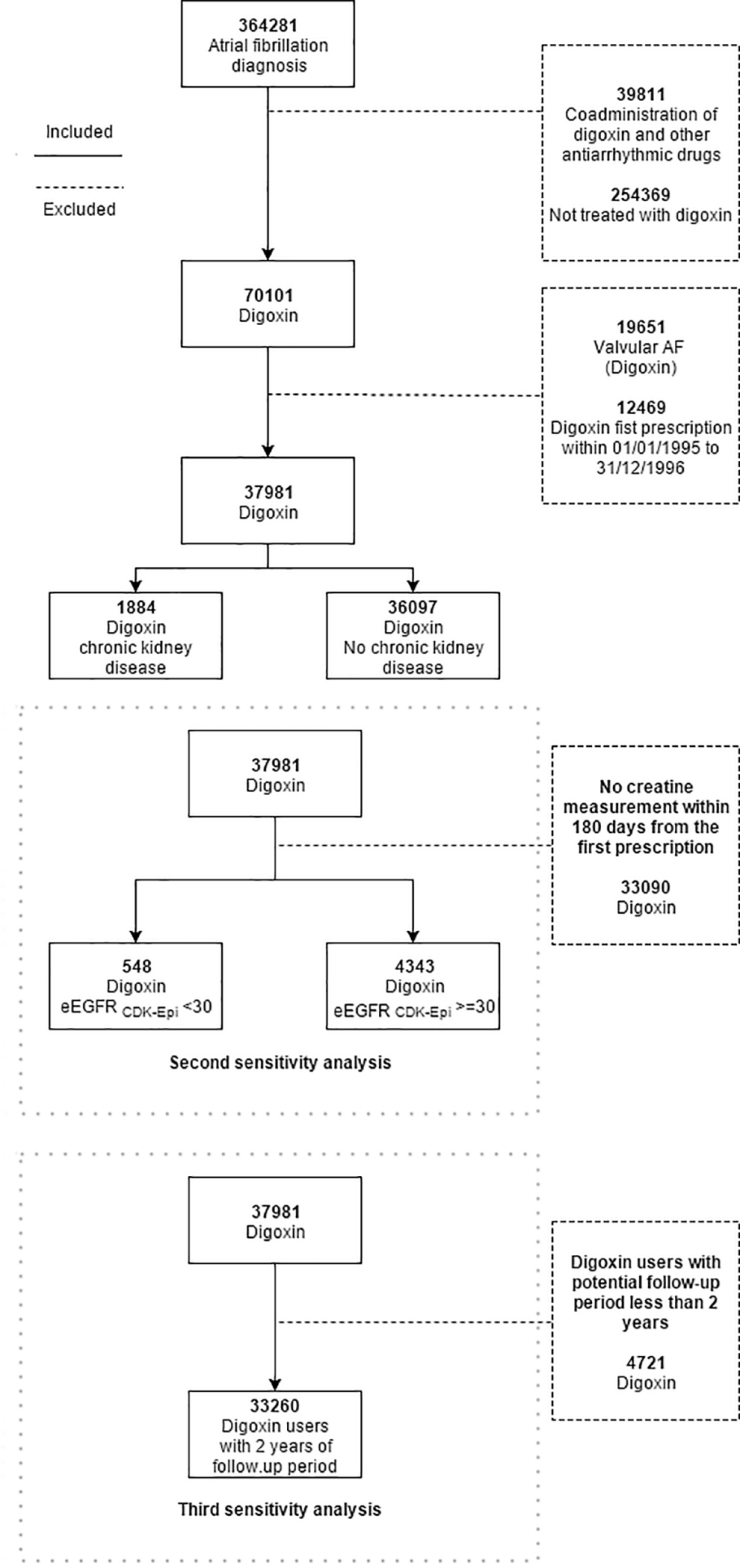
Flowchart. Flowchart of the study population and sensitivity analysis subpopulations.

**Fig 2:**
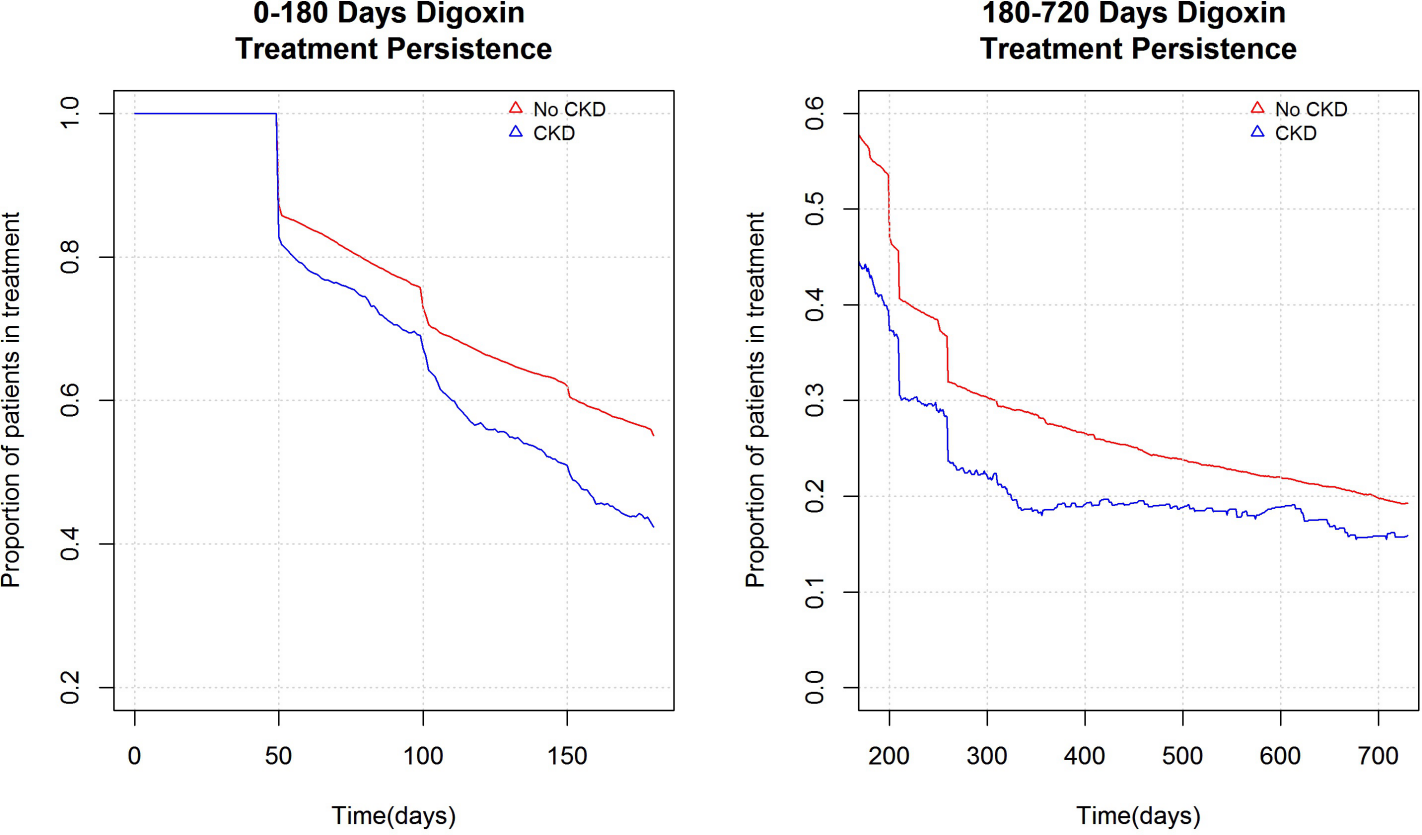
Persistence model. A treatment persistence model, showing the fraction of patients that is in treatment with digoxin on each relative day of the individual treatments periods.

We identified 37,981 patients with a diagnosis of atrial fibrillation that initiated digoxin therapy without simultaneous treatment with other antiarrhythmic drugs. Of these patients, 1884 patients had the diagnosis of chronic kidney disease (Fig 1). Within 180 days from index date, 54.7% of patients were in continuous digoxin treatment (Fig 2). Baseline characteristics of the patients are presented in Table 1. Patients with chronic kidney disease were younger and had more comorbidities compared to patients without chronic kidney disease. During the study period, only few patients had the measurement (4551 patients) of serum haemoglobin concentration 180 days before index date, resulting to have a mean serum haemoglobin concentration of 7g/dL. This sub-population was investigated to identify the major causes of this clinical condition, discovering that 1137 (24.98%) out of 4551 patients had diagnosis of anaemia (ICD10 codes: D60-D64). Of the 32947 patients with CHA2DS2-VASc score >1, 18201 (55.24%) patients were on antithrombotic treatment, of which 13028 patients were treated with low-dose aspirin (39.54%) and 617 (1.87%) on warfarin.

**Table 1:** Demographic characteristics of all patients (N = 37,981) with non-valvular atrial fibrillation and/or atrial flutter as hospitalization diagnosis from January 1, 1997 to December 31, 2012 identified in Danish nationwide administrative registries and treated with digoxin for these pathologies.

Variable	Level -N^b^(%) or Mean (SD^c^)	No CKD^a^ (N^b^ = 36,097)	CKD^a^ (N^b^ = 1884)	Total (N^b^ = 37,981)	p-value
Age in years–mean (SD^c^)	N^b^(%)	81.7 (10.6)	81.1 (9.7)	81.7 (10.5)	0.024
Sex (ref^d^. male)	N^b^(%)	17241 (47.8)	1134 (60.2)	18375 (48.4)	< 0.001
Alcohol abuse	N^b^(%)	1706 (4.7)	114 (6.1)	1820 (4.8)	0.010
Acute myocardial infarction		3762 (10.4)	287 (15.2)	4049 (10.7)	< 0.001
Diabetes mellitus	N^b^(%)	4660 (12.9)	689 (36.6)	5349 (14.1)	< 0.001
Arterial embolism and thrombosis	N^b^(%)	10240 (28.4)	580 (30.8)	10820 (28.5)	0.025
Pulmonary embolism	N^b^(%)	651 (1.8)	54 (2.9)	705 (1.9)	0.001
Heart failure	N^b^(%)	13065 (36.2)	1053 (55.9)	14118 (37.2)	< 0.001
Hypertension	N^b^(%)	12898 (35.7)	1115 (59.2)	14013 (36.9)	< 0.001
Cancer	N^b^(%)	7131 (19.8)	446 (23.7)	7577 (19.9)	< 0.001
COPD^e^	N^b^(%)	7226 (20.0)	521 (27.7)	7747 (20.4)	< 0.001
Liver disease	N^b^(%)	829 (2.3)	89 (4.7)	918 (2.4)	< 0.001
Peripheral arterial disease	N^b^(%)	2837 (7.9)	296 (15.7)	3133 (8.2)	< 0.001
Stroke	N^b^(%)	9017 (25.0)	500 (26.5)	9517 (25.1)	0.135
Syncope	N^b^(%)	2517 (7.0)	158 (8.4)	2675 (7.0)	0.022
Ventricular arrhythmias	N^b^(%)	240 (0.7)	16 (0.8)	256 (0.7)	0.418
Serum potassium concentration (mEq/L)					
	Mean (SD^c^)	4.2 (0.5)	4.4 (0.6)	4.2 (0.5)	< 0.001
	Patient without measurement within 180 days before index date	31623 (87.6)	1543 (81.9)	33166 (87.3)	< 0.001
Serum haemoglobin concentration (g/dL)					
	Mean (SD^c^)	7.8 (1.2)	7.3 (1.1)	7.8 (1.2)	< 0.001
	Patient without measurement within 180 days before index date	31864 (88.3)	1566 (83.1)	33430 (88.0)	< 0.001
Lipid modifying agents	N^b^(%)	2102 (5.8)	185 (9.8)	2287 (6.0)	< 0.001
Loop diuretic	N^b^(%)	16493 (45.7)	1269 (67.4)	17762 (46.8)	< 0.001
RASi^f^	N^b^(%)	7635 (21.2)	564 (29.9)	8199 (21.6)	< 0.001
Diabetes mellitus medications	N^b^(%)	3870 (10.7)	504 (26.8)	4374 (11.5)	< 0.001
Antithrombotic medications	Total	18962 (52.5)	1052 (55.8)	20014 (52.7)	0.005
	Low-dose aspirin	13070 (36.2)	744 (39.5)	13814 (36.4)	0.004
	Warfarin	713 (2.0)	27 (1.4)	740 (1.9)	0.115
COPD^e^ drugs	N^b^(%)	4875 (13.5)	264 (14.0)	5139 (13.5)	0.553
NSAID^g^	N^b^(%)	4936 (13.7)	263 (14.0)	5199 (13.7)	0.751
Stroke risk (CHA2DS2-VASc^h^score)					
	High stroke risk	31183 (86.4)	1764 (93.6)	32947 (86.7)	< 0.001
	Intermediate stroke risk	2513 (7.0)	82 (4.4)	2595 (6.8)	< 0.001
	Low stroke risk	2401 (6.7)	38 (2.0)	2439 (6.4)	< 0.001
Digoxin dosage (μg)	mean *(*SD^c^*)*	73.7 (44.5)	65.8 (24.6)	73.3 (43.8)	< 0.001

^a^ CKD = chronic kidney disease.

^b^ N. = number.

^c^ SD = standard deviation.

^d^ ref. = reference.

^e^ COPD = Chronic Obstructive Pulmonary Disease.

^f^ RASi = Renin Angiotensin System inhibitor.

^g^ NSAID = Non-Steroidal Anti-inflammatory Drugs.

^h^ CHA2DS2-VASc score (C = Congestive heart failure; H = Hypertension; A = Age; D = Diabetes; S = Stroke; V = Vascular disease; sc = Sex category).

**Fig 3:**
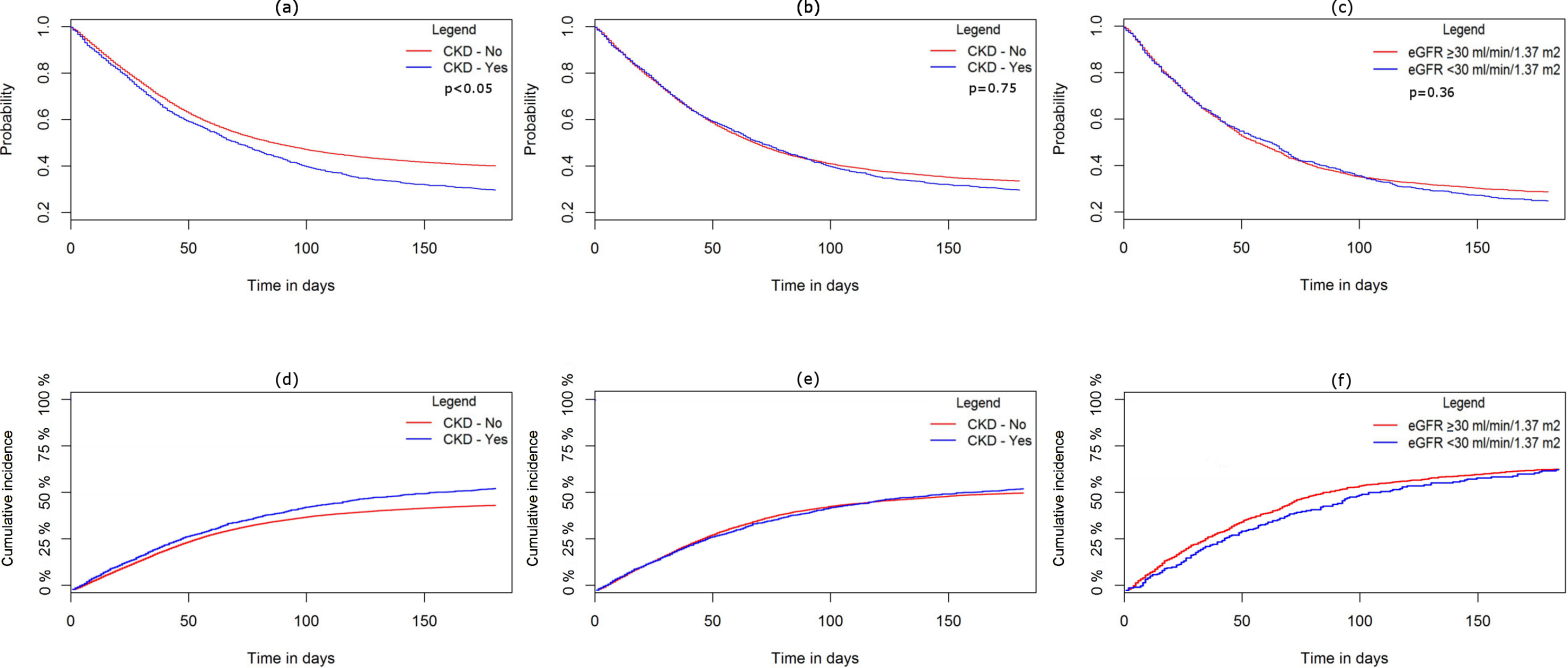
Survival curves. (a) Kaplan-Meier curve for all-cause mortality—main analysis–comparison among patients with and without chronic kidney disease. (b) Kaplan-Meier curve for all-cause mortality—first sensitivity analysis—comparison among patients with and without chronic kidney disease using a propensity-matched subpopulation. (c) Kaplan-Meier curve for all-cause mortality—second sensitivity analysis—comparison among patients with and without eGFR <30 ml/min/1.73m^2^ using a propensity-matched subpopulation. (d) Cumulative incidence for cardiovascular mortality—main analysis–comparison among patients with and without chronic kidney disease (Chi-square test statistic: 28.263; p-value < 0.001). (e) Cumulative incidence for cardiovascular mortality—first sensitivity analysis—comparison among patients with and without chronic kidney disease using a propensity-matched subpopulation. (f) Cumulative incidence for cardiovascular mortality—second sensitivity—analysis comparison among patients with and without eGFR <30 ml/min/1.73m^2^ using a propensity-matched subpopulation. Comparison among survival curves for all-causes was performed using log-rank test and the p-value was shown in the figure.

The study population of 37,981 patients was followed up for 10,294 person-year (median = 82 days; range = 0–180 days), 22,536 (59.3%) patients died within 180 days of follow-up period. Among these, 14,862 (39.1%) patients died for cardiovascular disease. Kaplan-Meier survival curves differed between the two cohorts for all-causes mortality (p<0.05) (Fig 3A) as like unadjusted Cox regression analysis showed an increased risk for all-causes and cardiovascular mortality among patients with chronic kidney disease (Table 2). However, Cox regression analysis adjusted for age, sex, year of inclusion, diagnosis of alcohol abuse, myocardial infarction, diabetes mellitus, heart failure, hypertension, cancer, COPD, liver disease, syncope, peripheral arterial disease, ventricular arrhythmia, serum haemoglobin and potassium concentration, stroke or systemic thromboembolism history, CHA2DS2-VASc score, digoxin dosage and drugs listed in S2 Table, revealed no statistically significant differences in all-causes [Hazard Ratio (HR) 0.89; 95% Confident Interval (CI) 0.78–1.03)] and cardiovascular mortality (HR 0.88; 95%CI 0.74–1.05) among patients with and without chronic kidney disease within 180 days from index date (Table 2). Several independent predictors for all-causes and cardiovascular mortality were identified, as showed in S3 Table. In particular, peripheral arterial disease was found associated to an increased risk of 17% (HR 1.17; 95%CI 1.04–1.32) for all causes mortality and 28% for cardiovascular mortality (HR 1.28; 95%CI 1.10–1.48). Heart failure was associated to an increased risk of 16% (HR 1.16 95%CI 1.07–1.26) for all causes mortality and 30% for cardiovascular mortality (HR 1.30; 95%CI 1.18–1.44). COPD and cancer was associated to an increased risk in all-causes mortality (S3 Table). Among administered drugs, loop diuretic and low-dose aspirin administration were associated to an increased risk of all-causes and cardiovascular mortality. Loop diuretic shown an increase of 24% in all-causes mortality (HR 1.24; 95%CI 1.14–1.34) and 26% in cardiovascular mortality (HR 1.26; 95%CI 1.14–1.40). Low dose aspirin showed an increase of 20% in all-causes mortality (HR 1.20; 95%CI 1.08–1.34) and 15% in cardiovascular mortality (HR 1.15; 95%CI 1.01–1.32). For both all-causes and cardiovascular mortality, warfarin administration shown no statistically significant increase in mortality between the two cohorts. Effect modification was found for age and CHA2DS2-VASc score for all-causes and cardiovascular mortality (p<0.05). In particular, there was a progressive increase in risk of both all-causes and cardiovascular mortality comparing patients with CHA2DS2-VASc <1 to patients with CHA2DS2-VASc = 1 or greater than one. Intermediate stroke risk (CHA2DS2-VASc score = 1) was associated to an increase in mortality of 21% for all-causes mortality and 102% for cardiovascular mortality compared to lower stroke risk. High stroke risk (CHA2DS2-VASc score>1) was associated to an increase in mortality of 92% for all-causes mortality and 306% for cardiovascular mortality compared to lower stroke risk. Similarly, for age, there was an increase in risk of both all-causes and cardiovascular mortality comparing age quartiles. Higher haemoglobin concentration in the two models showed a protective effect and effect modification (p<0.05) showing a progressive reduction in mortality comparing haemoglobin quartile. Lipid-modifying agents, Renin Angiotensin System inhibitor, antithrombotic therapy were protective factors for all-causes mortality, reducing the risk respectively by 21%, 13% and 14% (S3 Table).

**Table 2:** Summary of the unadjusted and adjusted Hazard Ratio for all-causes and cardiovascular mortality for all analysis.

		ALL CAUSES MORTALITY		CARDIOVASCULAR MORTALITY	
		Unadjusted Hazard Ratio (95% CI^a^)	Adjusted Hazard Ratio (95% CI^a^)	Unadjusted Hazard Ratio (95% CI^a^)	Adjusted Hazard Ratio (95% CI^a^)
**MAIN ANALYSIS**					
	CKD^b^ vs no-CKD^b^ 180 days follow-up period	1.26 (1.19–1.33)	0.89 (0.78–1.03)	1.24 (1.16–1.33)	0.88 (0.74–1.05)
**FIRST SENSITIVITY ANALYSIS**					
	CKD^b^ vs no-CKD^b^ 180 days follow-up period Propensity-match subpopulation	1.06 (0.99–1.13)	1.04 (0.98–1.10)	1.02 (0.95–1.10)	1.00 (0.93–1.08)
**SECOND SENSITIVITY ANALYSIS**					
	eGFR^c^ ≥30 vs No-eGFR^c^ ≥30 ml/min/1.73m2 180 days follow-up period Propensity-match subpopulation	1.02 (0.89–1.18)	0.97 (0.87–1.09)	0.97 (0.81–1.16)	1.00 (0.87–1.15)
**THIRD SENSITIVITY ANALYSIS**					
	**Part I**: CKD^b^ vs no-CKD^b^ 2 years follow-up period	1.32 (1.26–1.40)	0.90 (0.79–1.03)	1.31 (1.22–1.39)	0.87 (0.74–1.02)
	**Part II:** CKD^b^ vs no-CKD^b^ 2 years follow-up period Propensity-match subpopulation	1.09 (1.03–1.16)	1.06 (1.00–1.12)	1.04 (0.97–1.11)	1.00 (0.94–1.08)
	**Part III:** eGFR^c^ ≥30 vs No-eGFR^c^ ≥30 ml/min/1.73m2 2 years follow-up period Propensity-match subpopulation	1.10 (0.99–1.22)	1.00 (0.90–1.11)	1.13 (0.99–1.28)	1.00 (0.88–1.14)
**FOURTH SENSITIVITY ANALYSIS**					
	**180 days follow-up period**				
	eGFR^c^ (*as continuous variable*)	1.00 (0.99–1.00)	1.00 (1.00–1.01)	1.00 (0.99–1.00)	1.00 (0.99–1.00)
	Stage 4 (ref^d^. Stage *5*)	1.14 (0.76–1.25)	1.42 (0.96–2.09)	1.38 (0.98–1.93)	1.29 (0.73–2.27)
	Stage 3 (ref^d^. Stage *5*)	0.93 (0.73–1.18)	0.91 (0.72–1.16)	1.26 (0.91–1.75)	1.05 (0.75–1.46)
	Stage 2 (ref^d^. Stage *5*)	0.92 (0.72–1.16)	0.87 (0.68–1.10)	1.01 (0.79–1.52)	1.12 (0.80–1.55)
	Stage 1 (ref^d^. Stage *5*)	0.97 (0.76–1.25)	0.84 (0.65–1.08)	0.97 (0.56–1.67)	1.10 (0.78–1.55)
	**2 years follow-up period**				
	eGFR^c^ (*as continuous variable*)	1.00 (0.99–1.00)	1.00 (1.00–1.01)	1.00 (0.99–1.00)	1.00 (0.99–1.00)
	Stage 4 (ref^d^. Stage *5*)	1.02 (0.80–1.29)	1.39 (0.96–1.99)	1.41 (1.00–1.94)	1.07 (0.77–1.48)
	Stage 3 (ref^d^. Stage *5*)	0.91 (0.73–1.15)	0.90 (0.72–1.14)	1.25 (0.92–1.70)	1.08 (0.79–1.47)
	Stage 2 (ref^d^. Stage *5*)	0.93 (0.74–1.16)	0.84 (0.67–1.05)	1.13 (0.83–1.54)	1.03 (0.75–1.41)
	Stage 1 (ref^d^. Stage *5*)	1.05 (0.74–1.48)	0.84 (0.66–1.07)	1.08 (0.67–1.74)	1.46 (0.89–2.38)
**FIFTH SENSITIVITY ANALYSIS**					
	**180 days follow-up period**- CKD^b^ vs no-CKD^b^ Using narrow definition of CKD^b^	1.28 (1.17–1.40)	0.91 (0.75–1.11)	1.26 (1.14–1.40)	0.97 (0.76–1.23)
	**2 years follow-up period**—CKD^b^ vs no-CKD^b^ Using narrow definition of CKD^b^	1.35 (1.22–1.50)	0.91 (0.76–1.10)	1.35 (1.22–1.50)	0.92 (0.73–1.16)
**ADDITIONAL ANALYSIS**					
	**180 days follow-up period**- CKD^b^ vs no-CKD^b^ Using digoxin treatmentpersistence as time of exposure	1.28 (1.21–1.36)	0.91 (0.79–1.05)	1.27 (1.19–1.37)	0.89 (0.75–1.07)
	**2 years follow-up period**- CKD^b^ vs no-CKD^b^ Using digoxin treatmentpersistence as time of exposure	1.26 (1.19–1.33)	0.96 (0.84–1.10)	1.24 (1.16–1.33)	0.93 (0.79–1.09)

^a^ CI = Confident interval.

^b^ CKD = Chronic kidney disease.

^c^ eGFR = estimated Glomerular Filtration Rate.

^d^ ref. = reference.

### Chronic kidney disease vs. no-Chronic kidney disease and eGFR <30 vs eGFR ≥ 30 ml/min/1.73m^2^ comparison for all-causes and cardiovascular mortality using propensity-matched subpopulations

In the first sensitivity analysis, a comparison between patients with chronic kidney disease and patient without the pathology was performed for all-causes and cardiovascular mortality, using a propensity score matched subpopulation of 9420 patients (S4 Table). The subpopulation was followed for 2317 person-year (median = 66 days; range = 0–180 days) and 6201 (65.8%) patients died within 180 days of follow-up period. Among these, 4163 (44.2%) patients died for cardiovascular disease. Unadjusted Cox regression analysis showed no statistically significant increase in all-causes and cardiovascular mortality among patients with chronic kidney disease (Table 2). Propensity-matched multivariable Cox regression analysis revealed no significant statistical difference in all-causes (HR 1.04; 95%CI 0.98–1.10) and cardiovascular mortality (HR 1.00; 95%CI 0.93–1.08) comparing patients with and without chronic kidney disease (Table 2). In the second sensitivity analysis, a propensity score-matched subpopulation of 2740 patients was used to compare patients with and without eGFR < 30 ml/min/1.73m^2^ for all-causes and cardiovascular mortality (S5 Table). The subpopulation was followed up for 614 person-year (median = 56 days; range = 0–180 days) and 1938 (70.8%) patients died within 180 days of follow-up period. Among these, 1284 (46.9%) patients died for cardiovascular disease. Unadjusted Cox regression analysis showed no statistically significant increase in all-causes and cardiovascular mortality among patients with chronic kidney disease (Table 2). Propensity-matched multivariable Cox regression analysis revealed no significant statistical difference in all-causes (HR 0.97; 95%CI 0.87–1.09) and cardiovascular mortality (HR 1.00; 95%CI 0.87–1.15) comparing patients with and without eGFR <30 ml/min/1.73m^2^ (Table 2). Kaplan-Meier survival curves did not differ for all-causes mortality among cohorts for both sensitivity analyses (Fig 3B and 3C). The effects of each predictor for each sensitivity analysis were shown in S6 Table.

### Chronic kidney disease vs. no-Chronic kidney disease and eGFR <30 vs eGFR ≥ 30 ml/min/1.73m^2^ comparison for all-causes and cardiovascular mortality using a 2 years follow-up period

In the first part of the third sensitivity analysis, a comparison among patients with chronic kidney disease and patient without the pathology was performed for all-causes and cardiovascular mortality in a longer follow-up period of 2 years. A subpopulation of 33,260 patients with 2 years potential follow-up period was used. Adjusted analysis performed showed no statistically significant effect of chronic kidney disease on all-causes mortality (HR 0.90; 95%CI 0.79–1.03) and cardiovascular mortality (HR 0.87; 95%CI 0.74–1.02) despite unadjusted Cox regression analysis showed statistically significant increase in all-causes and cardiovascular mortality among patients with chronic kidney disease (Table 2). The subpopulation was followed up for 22,063 person-year (median = 242 days; range = 0–730 days) and 25,292 (76.0%) patients died within 2 years from index date. Among these, 17261 (51.9%) patients died for cardiovascular disease. Within 2 years from index date, 19.2% of patients were in continuous digoxin treatment (Fig 2). Among predictors, age and CHA2DS2-VASc score had an effect modification (p<0.05), resulting in an increased risk of all-causes and cardiovascular mortality increasing the score. In particular, there was an increase in risk of both all-causes and cardiovascular mortality comparing patients with CHA2DS2-VASc <1 to patients with CHA2DS2-VASc = 1 or greater than one. Patients with high stroke risk (CHA2DS2-VASc score>1) had an increased risk of 275% for cardiovascular mortality compared to those with low stroke risk (CHA2DS2-VASc score<1) and 104% for all-causes mortality (S3 Table). Among predictors for all-causes and cardiovascular mortality, acute myocardial infarction, peripheral arterial disease, COPD and loop diuretic administration resulted associated to an increased risk of death. Warfarin administration shown a non-statistical increase in all-causes and cardiovascular mortality. Instead, low-dose aspirin, showed an increased risk of all-causes mortality of 13% among users (HR 1.13; 95%CI 1.01–1.25). The effects of all other predictors was shown in S3 Table.

In the second part of the third sensitivity analysis, a comparison among patients with chronic kidney disease and patient without the pathology was performed for all-causes and cardiovascular mortality within 2 years from index date, using a propensity score matched subpopulation of 8195 patients (S7 Table). The subpopulation was followed up for 4296 person-year (median = 59 days; range = 0–730 days) and 6829 (83.3%) patients died within 2 years from index date. Among these, 4785 (58.4%) patients died for cardiovascular disease. Unadjusted Cox regression analysis showed statistically significant increase in all-causes mortality but not for cardiovascular mortality among patients with chronic kidney disease (Table 2). However, propensity-matched multivariable Cox regression analysis revealed no significant statistical difference in all-causes (HR 1.06; 95%CI 1.00–1.12) and cardiovascular mortality (HR 1.00; 95%CI 0.94–1.08) comparing patients with and without chronic kidney disease (Table 2). The effects of each predictor was shown in S8 Table.

In the third part of the third sensitivity analysis, a comparison between patients with eGFR <30 and those with eGFR ≥ 30 ml/min/1.73m^2^ was performed for all-causes and cardiovascular mortality within 2 years from index date, using a propensity score matched subpopulation of 2530 patients (S9 Table). The subpopulation was followed up for 1229 person-year (median = 52 days; range = 0–730 days) and 2132 (84.3%) patients died within 2 years from index date. Among these, 1459 (57.7%) patients died for cardiovascular disease. Unadjusted Cox regression analysis showed no statistically significant increase in all-causes and cardiovascular mortality among patients with chronic kidney disease (Table 2). Propensity-matched multivariable Cox regression analysis revealed no significant statistical difference in all-causes (HR 1.00; 95%CI 0.90–1.11) and cardiovascular mortality (HR 1.00; 95%CI 0.88–1.14) between the two cohorts (Table 2). The effects of each predictor for each sensitivity analysis was shown in S8 Table.

### eGFR as continuous or multi-categorical predictor for all-causes and cardiovascular mortality within 180 days and 2 years from index date

In the fourth sensitivity analysis, we assessed the risk of all causes and cardiovascular mortality on a propensity score-matched subpopulation of 2740 patients with creatinine measurement, using eGFR as continuous predictor and multi-categorical predictor for both 180 days and 2 years follow-up period (S5 Table). Unadjusted Cox regression analysis showed no statistically significant decrease in all-causes and cardiovascular mortality increasing eGFR for both 180 days and 2 years follow-up period (Table 2). Propensity-matched multivariable Cox regression analysis revealed no significant statistical decrease in all-causes mortality within 180 days (HR 1.00; 95%CI 1.00–1.01) and 2 years (HR 1.00; 95%CI 1.00–1.01) follow-up period, increasing eGFR. No statistically significant decrease in the risk cardiovascular mortality was found neither for 180 days (HR 1.00; 95%CI 0.99–1.00) nor for 2 years (HR 1.00; 95%CI 0.99–1.00) follow-up period, increasing eGFR. Unadjusted Cox regression analysis showed statistically no significant differences in all-causes and cardiovascular mortality among patients with different chronic kidney disease stages for both 180 days and 2 years follow-up period (Table 2). No statistically significant difference in all-causes mortality was found in the adjusted analysis within 180 days (reference group Stage 5; Stage 1 HR 0.84 95%CI 0.65–1.08; Stage 2 HR 0.87 95%CI 0.68–1.10; Stage 3 HR 0.91 95%CI 0.72–1.16; Stage 4 HR 1.42 95%CI 0.96–2.09) follow-up period. Similar results was found using a 2 years follow-up period (reference group Stage 5; Stage 1 HR 0.84 95%CI 0.66–1.07; Stage 2 HR 0.84 95%CI 0.67–1.05; Stage 3 HR 0.90 95%CI 0.72–1.14; Stage 4 HR 1.39 95%CI 0.96–1.99). Similarly, no statistically significant difference was found for cardiovascular mortality within 180 days (reference group Stage 5; Stage 1 HR 1.10 95%CI 0.78–1.55; Stage 2 HR 1.12 95%CI 0.80–1.55; Stage 3 HR 1.05 95%CI 0.75–1.46; Stage 4 HR 1.29 95%CI 0.73–2.27) follow-up period. Neither using a 2 years follow-up period (reference group Stage 5; Stage 1 HR 1.46 95%CI 0.89–2.38; Stage 2 HR 1.03 95%CI 0.75–1.41; Stage 3 HR 1.08 95%CI 0.79–1.47; Stage 4 HR 1.07 95%CI 0.77–1.48).

### Comparison for all-causes and cardiovascular mortality within 180 days and 2 years from index date using a narrow definition of chronic kidney disease

In the fifth sensitivity analysis, we used a narrow definition of chronic kidney disease. Using this definition, among 37,981 patients with a diagnosis of atrial fibrillation that initiated digoxin therapy, 725 patients had the diagnosis of chronic kidney disease compared to 1,884 obtained using a broad definition (S10 Table). Of these patients, 33,260 had a 2-years potential follow-up period, of which 608 patients had the diagnosis of chronic kidney disease. Unadjusted Cox regression analysis showed statistically significant increase in all-causes and cardiovascular mortality among patients with chronic kidney disease for both 180 days and 2 years follow-up period (Table 2). However, adjusted Cox regression analysis revealed no significant statistical difference in all-causes mortality within 180 days (HR 0.91; 95%CI 0.75–1.11) and 2 years (HR 0.91; 95%CI 0.76–1.10) follow-up period among patients with and without chronic kidney disease (Table 2). Similarly, no significant statistical difference in cardiovascular mortality was found within 180 days (HR 0.97; 95%CI 0.76–1.23) and 2 years (HR 0.92; 95%CI 0.73–1.16) follow-up period (Table 2).

### Comparison for all-causes and cardiovascular mortality using the persistence of digoxin treatment to determine the time of exposure to digoxin

In the additional analysis, we assessed the risk of all-causes and cardiovascular mortality using digoxin treatment persistence to determine the time of exposure to digoxin within 180 days and 2 years follow-up period from the first digoxin prescription. In the cohort of 37,981 patients with a diagnosis of atrial fibrillation that initiated digoxin therapy, within 180 days follow-up period, adjusted Cox regression analysis showed no statistically significant increase in all-causes (HR 0.91; 95%CI 0.79–1.05) and cardiovascular mortality (HR 0.89; 95%CI 0.75–1.07) among patients with chronic kidney disease within 180 days follow-up period (Table 2).

Similarly, within a 2 years follow-up period, adjusted Cox regression analysis showed no statistically significant increase in all-causes (HR 0.96; 95%CI 0.84–1.10) and cardiovascular mortality (HR 0.93; 95%CI 0.79–1.09) among patients with chronic kidney disease within 2 years follow-up period (Table 2). Unadjusted and adjusted hazard ratio for both all-causes and cardiovascular mortality within 180 days and 2 years follow-up period were shown in Table 2.

## Discussion

To our knowledge, this is the first observational study that directly examined the effect of chronic kidney disease and its stages on all-causes and cardiovascular mortality among patients treated with digoxin as unique antiarrhythmic agent for non-valvular atrial fibrillation. To date, only one study indirectly tried to evaluate the effect of renal function on mortality in a subpopulation of patients with atrial fibrillation, showing a non-statistically significant interaction term of renal function on mortality in the comparison between digoxin and non-digoxin users [13]. The current study, instead, directly compared patients with and without chronic kidney disease, and patients with different stages of chronic kidney disease for all-causes and cardiovascular mortality, among patients treated with digoxin as unique antiarrhythmic agent for non-valvular atrial fibrillation. This study design was chosen to avoid the effect of indication bias in the evaluation of effect of chronic kidney disease on mortality comparing patients treated with different heart rate control agents. We believe this was necessary because the use of a specific heart rate control agent was a choice made by the cardiologist based on the clinical judgement, after an appropriate examination of the patient and it is not a random event. An unknown number of factors may influence the physician prescription of antiarrhythmic drug including clinical or prescriber-related factors (recorded or not). Therefore, potential unknown factors could influence this association comparing patients treated with different antiarrhythmic drugs. This paper, therefore contribute to the current literature both by providing the results of a direct evaluation of the effect of chronic kidney disease on all-causes and cardiovascular mortality, both by reducing the effect of indication bias in this evaluation, among patients treated with digoxin as unique antiarrhythmic agent for non-valvular atrial fibrillation. Moreover, our study provide new information to the literature regarding the direct effect of chronic kidney disease stages in all-causes and cardiovascular mortality among patients treated with digoxin as unique antiarrhythmic agent for non-valvular atrial fibrillation. Recently, increased awareness has emerged regarding the effect of the chronic kidney disease stages on clinical outcome, especially among cardiologists treating arrhythmic disorders [29]. The main reason is related to the complexity of the pathological and physiological interactions between the kidney and the heart that could have clinical implications. However, few information are available on this topic and little is known regarding how different antiarrhythmic drugs could potentially change these interactions, especially for drugs with narrow therapeutic ranges like digoxin [47]. Our results shown that no statistically significant difference exist among patients with eGFR <30 ml/min/1.73m^2^ and those with eGFR ≥ 30 ml/min/1.73m^2^ or among different chronic kidney disease stages, in patients treated with digoxin as unique antiarrhythmic agent for non-valvular atrial fibrillation, within 180 days and 2 years from first digoxin prescription.

Despite not being able to explain any causal relationship for all these findings, we believe that a plausible explanation could be found in the pathophysiology of chronic kidney disease. Patients with chronic kidney disease have an excess in cardiovascular mortality because of a wide range of specific cardiovascular comorbidities. These includes heart failure, stroke, atherosclerosis, anaemia, peripheral artery disease, coronary disease and atrial fibrillation, which all have shared risk factors with chronic kidney disease [48]. In the current study, all the statistical models were adjusted for all these risk factors for all-causes and cardiovascular mortality and consequently, the effect of the chronic kidney disease on mortality was evaluated without the effect of its related comorbidities showing a not statistically significant direct effect on mortality. Reinforcing this hypothesis, in the current study the major impact on mortality was mainly found to be associated with comorbidities commonly related to chronic kidney disease [48]. In particular, the presence of heart failure, peripheral arterial disease and anaemia. Moreover, we believe that another possible explanation for our results is that in our study we also adjusted all the statistical models for digoxin dosage. Digoxin is mainly eliminated through renal excretion, which is closely related to renal function [49]. Changes in renal function due to chronic kidney disease could drastically change digoxin serum concentration [50]. It is well accepted, that digoxin benefit/risk ratio (including mortality) is highly related to its dosage and consequently to its serum concentration. Digoxin has a narrow therapeutic window and even small changes in dosage could affect changes in the risk of toxicity. Several pieces of evidence suggest that increasing digoxin dosage and its related serum concentration does not increase the clinical effectiveness. Higher dosage provides calcium loading that does not increase therapeutic effects, and may cause harm by calcium overload [14,28,51–53]. However, in our model no direct effect of dosage on all-causes and cardiovascular mortality was observed. A plausible explanation is that patient with chronic kidney disease were monitored carefully regarding digoxin posology. Reinforcing this hypothesis, in our study population a lower digoxin dosage was found among patients with chronic kidney disease.

We believe that another plausible explanation for our results is that all the comparison among cohorts for all-causes and cardiovascular mortality was performed adjusting for the co-administrated drug with potential negative effect on mortality in this study population. In fact, it is interesting to denote that in our study co-administration of loop diuretic and low-dose aspirin shown an increased risk of all causes and cardiovascular mortality in this study population. These results were not surprising considering that both loop diuretics and low-dose aspirin have adverse effects on kidney function, and that loop diuretic could give pharmacokinetic interactions with digoxin, increasing the risk of adverse clinical outcome [54–63]. However, the administration of these drugs could also be a proxy for a worst clinical condition. Considering that safety evaluation in these subpopulations was not an aim of this study, no specific sub-analysis was performed on these subpopulations. Therefore, more studies are required on this topic to clarify these associations. Finally, we believe that another possible explanation for our results is that all the statistical models were adjusted for drugs with positive survival effect in atrial fibrillation patients, like lipid-modifying agents [64,65]. In particular, is well-know that exist a higher risk of atherosclerosis among patients with chronic kidney disease and the lipid-modifying agents in this subpopulation shown a protective effects on atherosclerosis and cardiovascular mortality [64,65]. In fact, our results shown that the administration of lipid modifying agents shown instead a protective effect on both all-causes and cardiovascular mortality. However, considering that safety evaluation in these subpopulations was not an aim of this study, no specific sub-analysis was performed on these subpopulations. Therefore, more studies are required on this topic to clarify these associations.

## Conclusion

This study suggest no direct effect of chronic kidney disease and also chronic kidney disease stages on all-causes and cardiovascular mortality within 180 days and 2 years from the first prescription of digoxin among patients treated with digoxin for non-valvular atrial fibrillation.

Despite more studies are necessary to clarify this association, based on our results, whenever the clinical conditions suggested the use of digoxin as unique antiarrhythmic treatment for atrial fibrillation, we suggested that the presence of chronic kidney disease should not represent an obstacle for the prescription of digoxin if a renal excretion-based adjustment of the dosage is performed. As well as a careful monitoring of the sign and symptoms of digoxin intoxication.

## Limitation

The evaluation of the effect of chronic kidney disease and its stages on all-causes and cardiovascular mortality was performed using an intention to treat approach. This approach could not account for a possible change in the treatment or covariate during the follow-up period, especially in the longer follow-up period. A limited amount of patients in our population had biomarkers measurement (e.g. creatinine, potassium, etc.) within 180 days before index date potentially due to the data-source used. The LABKA database cover 1.8 million people for a period of more than 10 years only from the North Denmark region and the Central Denmark region. Other limitations included a low prevalence of patients with chronic kidney disease compared to those expected in end-of-life population of patients with atrial fibrillation, short median observation period of 82 days and low persistence in digoxin treatment. Moreover, another limitation include a lower prevalence of patients in treatment with warfarin (1.9%) to those expected in patients with atrial fibrillation. These limitations should be considered when interpreting the results.

## Supporting Information

S1 Table Diagnoses, surgical procedures, and medicines used for defining the population and comorbidity. (DOCX)

S2 Table Co-administrated drugs considered. (DOCX)

S3 Table CKD vs no-CKD all-causes and cardiovascular mortality comparison within 180 days follow-up period. (DOCX)

S4 Table First sensitivity analysis propensity score matched subpopulation (N = 9420). (DOCX)

S5 Table Second sensitivity analysis propensity score matched subpopulation (N = 2740). (DOCX)

S6 Table CKD vs no-CKD and eGFR <30 vs eGFR ≥ 30 ml/min/1.73m2 comparison for all-causes and cardiovascular mortality within 180 days follow-up period using propensity-matched subpopulations. (DOCX)

S7 Table Third sensitivity analysis–part 2 propensity score matched subpopulation (N = 8195). (DOCX)

S8 Table CKD vs no-CKD and eGFR <30 vs eGFR ≥ 30 ml/min/1.73m2 comparison for all-causes and cardiovascular mortality within 2 years follow-up period using propensity-matched subpopulations. (DOCX)

S9 Table Third sensitivity analysis–part 3 propensity score matched subpopulation (N = 2530). (DOCX)

S10 Table Fifth sensitivity analysis population (N = 37,981). (DOC)
