# Supplementary material for: Effect of Chronic Kidney Diseases on Mortality among Digoxin Users Treated for Non-Valvular Atrial Fibrillation: A Nationwide Register-Based Retrospective Cohort Study
Source: PLoS One. 2016 Jul 28;11(7):e0160337. doi: 10.1371/journal.pone.0160337 (PMC4965154; doi:10.1371/journal.pone.0160337)
Supplement: S1 Table — (DOCX) [file pone.0160337.s001.docx]

**S1 Table. Diagnoses, surgical procedures, and medicines used for defining the population and comorbidity.**

| **Non-valvular atrial fibrillation** | |
| --- | --- |
| Presence of | *ICD10* ^a^: I48  *ICD8* ^b^: 42793-42794 |
| Absence of | *ICD10* ^a^: I05, I06, I34, I35  *ICD8* ^b^: 394-396, 4240, 4241  *NCSP* ^c^: KFK, KFM, KFP |

| **Comorbidity** | | |
| --- | --- | --- |
| Alcohol abuse | Defined from diagnosis and adverse alcohol consumption reported during hospitalization | *ICD10*^a^: E244, E52, F10, G312, G621, G721, I426, K292, K70, K860, L278A, O354, T51, Z714, Z721 |
| Chronic kidney disease | Defined from diagnosis | *ICD10*^a^: E102, E112, E132, E142, I120, M3215, M300, M313, M319, N02-N08, N11-N12, N14, N158-N160, N162-N164, N168, N18-N19, N26, Q612-Q613, Q615, Q619, |
| Chronic obstructive pulmonary disease (COPD) | Defined from treatment and diagnosis | Bronchial dilating medication for inhalation (ATC^d^ code R03) and/or an admission for a chronic obstructive pulmonary disorder (J42-44) |
| Diabetes mellitus | Defined from medicines | *Treatment*: Glucose-lowering medication |
| Heart failure | Defined from diagnosis | *ICD10*^a^: I110, I42, I50, J819 |
| Hypertension | Defined from combination treatment with a least two classes of antihypertensive drugs. This definition of hypertension has a positive predictive value of 80.0% and a specificity of 94.7%. | *Treatment*: Adrenergic α-antagonist, non-loop-diuretics, vasodilators, beta blockers, calcium channel blockers, and renin-angiotensin system inhibitors. |
| Liver disease | Defined from diagnoses of liver cancer, chronic liver disease, liver surgery, cirrhosis, and hepatitis | *ICD10*^a^: B15-B19, C22, D684C, I982B, K70-K77, Z944 |
| Myocardial infarction | Defined from diagnosis | *ICD10*^a^: I21-I22 |
| Peripheral artery disease | Defined from diagnosis | *ICD10*^a^: I700, I702, I709 |
| Syncope | Defined from diagnoses | *ICD10*^a^: R55 |
| Stroke or systemic thromboembolism history | Defined from diagnoses of peripheral artery embolism, stroke, and transient ischaemic attack | *ICD10*^a^: G458, G459, I63, I64, I74 |
| Ventricular arrhythmia | Defined from diagnoses | *ICD10*^a^: I472, I490 |

^a^ICD10: 10^th^ revision of the International Classification of Diseases system

^b^ICD8: 8^th^ revision of the International Classification of Diseases system

^c^NCSP: The Nordic Medical Statistics Committees Classification of Surgical Procedures

^d^ATC: Anatomical Therapeutic Classification
