# Supplementary material for: Effect of Chronic Kidney Diseases on Mortality among Digoxin Users Treated for Non-Valvular Atrial Fibrillation: A Nationwide Register-Based Retrospective Cohort Study
Source: PLoS One. 2016 Jul 28;11(7):e0160337. doi: 10.1371/journal.pone.0160337 (PMC4965154; doi:10.1371/journal.pone.0160337)
Supplement: S2 Table — (DOCX) [file pone.0160337.s002.docx]

**S2 Table. Co-administrated drugs considered.**

| **Anatomical Therapeutic Classification (ATC) codes** | |
| --- | --- |
| Loop diuretics  Digoxin  Glucose lowering medication  Low-dose aspirin  Antithrombotic agents  Lipid modifying agents  Non-steroidal anti-inflammatory drugs (NSAIDs)  Warfarin  Renin Angiotensin System-inhibitor (RAS-i)  Drugs for COPD^a^ | C03C  C01AA05  A10  B01AC06  B01  C10  M01A  B01AA03  C09  R03 |

^a^COPD = Chronic Obstructive Pulmonary Disease.
