# Supplementary material for: Effect of Chronic Kidney Diseases on Mortality among Digoxin Users Treated for Non-Valvular Atrial Fibrillation: A Nationwide Register-Based Retrospective Cohort Study
Source: PLoS One. 2016 Jul 28;11(7):e0160337. doi: 10.1371/journal.pone.0160337 (PMC4965154; doi:10.1371/journal.pone.0160337)
Supplement: S3 Table — (DOCX) [file pone.0160337.s003.docx]

**S3 Table. CKD vs no-CKD all-causes and cardiovascular mortality comparison within 180 days follow-up period.**

|  | **MAIN ANALYSIS  CKD**^a^ **(N=1884) vs no-CKD**^a^ **(N=36,097) 180 Days Follow-up Total = 37,981** | | **THIRD SENSITIVITY ANALYSIS PART 1**  **CKD**^a^ **(N=1639) vs no-CKD**^a^ **(N=31,621) 2 Years Follow-up Total = 33,260** | |
| --- | --- | --- | --- | --- |
|  | **Hazard Ratio (95%  Confident interval)** | | **Hazard Ratio (95%  Confident interval)** | |
|  | ***All-causes mortality*** | ***Cardiovascular mortality*** | ***All-causes mortality*** | ***Cardiovascular Mortality*** |
| Chronic kidney disease | 0.89 (0.78-1.03) | 0.88 (0.74-1.05) | 0.90 (0.79-1.03) | 0.87 (0.74-1.02) |
| Age quartile | *ref*^b^*. quartile 1* | *ref*^b^*. quartile 1* | *ref*^b^*. quartile 1* | *ref*^b^*. quartile 1* |
| Age quartile 2 | 1.20 (1.05-1.36) | 1.26 (1.07-1.49) | 1.30 (1.15-1.46) | 1.36 (1.17-1.59) |
| Age quartile 3 | 1.45 (1.27-1.66) | 1.67 (1.41-1.98) | 1.55 (1.36-1.76) | 1.36 (1.17-1.59) |
| Age quartile 4 | 1.74 (1.52-2.00) | 2.06 (1.73-2.46) | 1.94 (1.70-2.22) | 2.32 (1.97-2.75) |
| Sex (*ref*^b^*. Male*) | 1.04 (0.97-1.12) | 1.07 (0.98-1.18) | 1.05 (0.97-1.13) | 1.07 (0.98-1.17) |
| Year of inclusion | *ref*^b^*. 1997 to 2000* | *ref*^b^*. 1997 to 2000* | *ref*^b^*. 1997 to 2000* | *ref*^b^*. 1997 to 2000* |
| 2001 to 2004 | 1.25 (1.12-1.39) | 1.24 (1.10-1.41) | 1.11 (1.01-1.22) | 1.08 (0.96-1.21) |
| 2005 to 2008 | 1.38 (1.23-1.55) | 1.20 (1.04-1.38) | 1.21 (1.09-1.34) | 1.03 (0.90-1.17) |
| 2009 to 2012 | 0.82 (0.72-0.94) | 0.55 (0.46-0.65) | 1.12 (0.98-1.28) | 0.89 (0.75-1.06) |
| Alcohol abuse | 1.11 (0.93-1.31) | 0.96 (0.76-1.22) | 1.17 (0.99-1.38) | 1.05 (0.84-1.32) |
| Acute myocardial infarction | 1.08 (0.97-1.21) | 1.10 (0.96-1.26) | 1.12 (1.01-1.24) | 1.14 (1.01-1.29) |
| Diabetes mellitus | 1.09 (0.95-1.25) | 1.16 (0.98-1.37) | 1.13 (0.99-1.29) | 1.19 (1.01-1.39) |
| Arterial thrombosis | 1.09 (0.90-1.32) | 1.22 (0.96-1.54) | 1.08 (0.90-1.30) | 1.19 (0.96-1.48) |
| Pulmonary thrombosis | 1.01 (0.76-1.33) | 0.94 (0.67-1.32) | 1.11 (0.86-1.43) | 1.11 (0.83-1.49) |
| Heart failure | 1.16 (1.07-1.26) | 1.30 (1.18-1.44) | 1.20 (1.11-1.30) | 1.30 (1.18-1.44) |
| Hypertension | 0.96 (0.89-1.04) | 0.96 (0.87-1.06) | 0.99 (0.91-1.07) | 1.01 (0.92-1.11) |
| Cancer | 1.33 (1.22-1.44) | 0.96 (0.86-1.07) | 1.35 (1.25-1.47) | 0.99 (0.89-1.10) |
| COPD^c^ | 1.25 (1.13-1.38) | 1.11 (0.98-1.26) | 1.26 (1.15-1.39) | 1.14 (1.01-1.28) |
| Liver disease | 1.01 (0.81-1.26) | 0.87 (0.63-1.20) | 1.07 (0.85-1.33) | 0.96 (0.71-1.30) |
| Peripheral arterial disease | 1.17 (1.04-1.32) | 1.28 (1.10-1.48) | 1.15 (1.02-1.29) | 1.27 (1.11-1.46) |
| Stroke | 1.16 (0.96-1.42) | 1.17 (0.93-1.49) | 1.19 (0.99-1.44) | 1.19 (0.96-1.48) |
| Syncope | 1.07 (0.94-1.22) | 1.16 (0.99-1.36) | 1.12 (0.99-1.27) | 1.21 (1.04-1.40) |
| Ventricular arrhythmias | 0.96 (0.64-1.44) | 1.15 (0.73-1.81) | 0.99 (0.67-1.45) | 1.10 (0.71-1.70) |
| Serum potassium (mEq/L) | 0.93 (0.86-1.00) | 0.97 (0.88-1.06) | 0.96 (0.90-1.04) | 1.00 (0.91-1.09) |
| Serum haemoglobin quartile | *ref*^b^*. quartile 1* | *ref*^b^*. quartile 1* | *ref*^b^*. quartile 1* | *ref*^b^*. quartile 1* |
| Haemoglobin quartile 2 | 0.81 (0.74-0.89) | 0.89 (0.79-1.01) | 0.87 (0.79-0.95) | 0.92 (0.82-1.03) |
| Haemoglobin quartile 3 | 0.76 (0.68-0.84) | 0.89 (0.78-1.01) | 0.77 (0.70-0.85) | 0.89 (0.79-1.01) |
| Haemoglobin quartile 4 | 0.59 (0.51-0.68) | 0.75 (0.63-0.89) | 0.63 (0.55-0.72) | 0.76 (0.64-0.89) |
| Haemoglobin NA^d^ | 0.77 (0.68-0.88) | 0.89 (0.76-1.05) | 0.79 (0.70-0.90) | 0.88 (0.76-1.03) |
| Lipid modifying agents | 0.79 (0.68-0.92) | 0.83 (0.69-1.00) | 0.77 (0.67-0.90) | 0.77 (0.64-0.92) |
| Loop diuretic | 1.24 (1.14-1.34) | 1.26 (1.14-1.40) | 1.22 (1.13-1.32) | 1.24 (1.12-1.36) |
| RASi^e^ | 0.87 (0.79-0.95) | 0.97 (0.87-1.09) | 0.86 (0.78-0.94) | 0.94 (0.84-1.04) |
| Low dose aspirin | 1.20 (1.08-1.34) | 1.15 (1.01-1.32) | 1.13 (1.01-1.25) | 1.08 (0.95-1.23) |
| Warfarin | 1.41 (0.98-2.05) | 1.48 (0.99-2.23) | 1.25 (0.88-1.78) | 1.30 (0.88-1.91) |
| Diabetes mellitus medication | 1.02 (0.88-1.19) | 1.02 (0.85-1.23) | 1.03 (0.89-1.20) | 1.06 (0.89-1.26) |
| Antithrombotic therapy | 0.86 (0.77-0.96) | 0.99 (0.86-1.13) | 0.93 (0.84-1.04) | 1.08 (0.95-1.23) |
| COPD^c^ drugs | 1.05 (0.94-1.19) | 1.02 (0.88-1.19) | 1.12 (1.00-1.25) | 1.06 (0.91-1.22) |
| NSAIDs^f^ | 1.02 (0.92-1.12) | 0.95 (0.84-1.08) | 1.02 (0.93-1.11) | 0.98 (0.87-1.10) |
| Stroke risk (CHA2DS2-VASc^g^ score) | *ref*^b^*. low stroke risk* | *ref*^b^*. low stroke risk* | *ref*^b^*. low stroke risk* | *ref*^b^*. low stroke risk* |
| High stroke risk | 1.92 (1.46-2.52) | 4.06 (2.48-6.65) | 2.04 (1.59-2.63) | 3.75 (2.47-5.69) |
| Medium stroke risk | 1.21 (0.89-1.64) | 2.02 (1.17-3.48) | 1.33 (1.01-1.75) | 1.91 (1.21-3.04) |
| Digoxin dosage (µg) | 1.00 (1.00-1.00) | 1.00 (1.00-1.00) | 1.00 (1.00-1.00) | 1.00 (1.00-1.00) |

Hazard Ratio and Confident Interval was estimated using multivariate Cox proportional hazard analysis adjusted for age, sex, year of inclusion, diagnosis of alcohol abuse, myocardial infarction, diabetes mellitus, heart failure, hypertension, cancer, chronic obstructive pulmonary disease, liver disease, syncope, peripheral arterial disease, ventricular arrhythmia, serum haemoglobin and potassium concentration, systemic thromboembolism history and CHA2DS2-VASc score. Moreover, the model was adjusted for digoxin dosage and co-administrated drugs.

^a^CKD = chronic kidney disease. ^b^ref. = reference. ^c^COPD = Chronic Obstructive Pulmonary Disease. ^d^NA = Not available. ^e^RASi = Renin Angiotensin System inhibitor. ^f^NSAID = Non-Steroidal Anti-inflammatory Drugs. ^g^CHA2DS2-VASc score (C = Congestive heart failure; H = Hypertension; A = Age; D = Diabetes; S = Stroke; V = Vascular disease; sc = Sex category).
