# Supplementary material for: Effect of Chronic Kidney Diseases on Mortality among Digoxin Users Treated for Non-Valvular Atrial Fibrillation: A Nationwide Register-Based Retrospective Cohort Study
Source: PLoS One. 2016 Jul 28;11(7):e0160337. doi: 10.1371/journal.pone.0160337 (PMC4965154; doi:10.1371/journal.pone.0160337)
Supplement: S4 Table — (DOCX) [file pone.0160337.s004.docx]

**S4 Table. First sensitivity analysis propensity score matched subpopulation (N=9420).**

| **Variable N**^b^ **(%) or Mean (SD**^c^**)** | **No CKD**^a^ **(N**^b^**=7536)** | | **CKD**^a^ **(N**^b^**=1884)** | **Total  (N**^b^**=9420)** | **p-value** | |
| --- | --- | --- | --- | --- | --- | --- |
| Age in years – *mean (SD*^c^*)* | | 81.5 (9.8) | 81.1 (9.7) | 81.4 (9.8) | | 0.134 |
| Sex (*ref^d^. male*) | | 4431 (58.8) | 1134 (60.2) | 5565 (59.1) | | 0.283 |
| Year of inclusion | |  |  |  | |  |
| 1997 to 2000 | | 2152 (28.6) | 521 (27.7) | 2673 (28.4) | |  |
| 2001 to 2004 | | 2026 (26.9) | 513 (27.2) | 2539 (27.0) | |  |
| 2005 to 2008 | | 1820 (24.2) | 458 (24.3) | 2278 (24.2) | |  |
| 2009 to 2012 | | 1538 (20.4) | 392 (20.8) | 1930 (20.5) | | 0.889 |
| Alcohol abuse | | 446 (5.9) | 114 (6.1) | 560 (5.9) | | 0.870 |
| Acute myocardial infarction | | 1121 (14.9) | 287 (15.2) | 1408 (14.9) | | 0.723 |
| Diabetes mellitus | | 2672 (35.5) | 689 (36.6) | 3361 (35.7) | | 0.381 |
| Arterial thrombosis | | 2344 (31.1) | 580 (30.8) | 2924 (31.0) | | 0.811 |
| Pulmonary thrombosis | | 210 (2.8) | 54 (2.9) | 264 (2.8) | | 0.913 |
| Heart failure | | 4201 (55.7) | 1053 (55.9) | 5254 (55.8) | | 0.929 |
| Hypertension | | 4340 (57.6) | 1115 (59.2) | 5455 (57.9) | | 0.220 |
| COPD^e^ | | 2004 (26.6) | 521 (27.7) | 2525 (26.8) | | 0.367 |
| Liver disease | | 340 (4.5) | 89 (4.7) | 429 (4.6) | | 0.739 |
| Peripheral arterial disease | | 1092 (14.5) | 296 (15.7) | 1388 (14.7) | | 0.193 |
| Stroke | | 2039 (27.1) | 500 (26.5) | 2539 (27.0) | | 0.672 |
| Syncope | | 645 (8.6) | 158 (8.4) | 803 (8.5) | | 0.846 |
| Ventricular Arrhythmias | | 61 (0.8) | 16 (0.8) | 77 (0.8) | | 0.977 |
| Lipid modifying agents | | 695 (9.2) | 185 (9.8) | 880 (9.3) | | 0.452 |
| Loop diuretic | | 5043 (66.9) | 1269 (67.4) | 6312 (67.0) | | 0.738 |
| RASi^f^ | | 2279 (30.2) | 564 (29.9) | 2843 (30.2) | | 0.818 |
| Low dose aspirin | | 2963 (39.3) | 744 (39.5) | 3707 (39.4) | | 0.912 |
| Warfarin | | 112 (1.5) | 27 (1.4) | 139 (1.5) | | 0.949 |
| Diabetes mellitus medication | | 1937 (25.7) | 504 (26.8) | 2441 (25.9) | | 0.368 |
| Antithrombotic therapy | | 4185 (55.5) | 1052 (55.8) | 5237 (55.6) | | 0.832 |
| COPD^e^ drugs | | 1016 (13.5) | 264 (14.0) | 1280 (13.6) | | 0.573 |
| NSAIDs^g^ | | 1055 (14.0) | 263 (14.0) | 1318 (14.0) | | 0.994 |
| CHA2DS2VASc^h^– *mean (SD*^b^*)* | | 5.7 (2.3) | 5.6 (2.3) | 5.6 (2.3) | | 0.544 |
| Stroke risk (CHA2DS2-VASc^h^ score) | |  |  |  | |  |
| High stroke risk | | 7063 (93.7) | 1764 (93.6) | 8827 (93.7) | |  |
| Medium stroke risk | | 323 (4.3) | 82 (4.4) | 405 (4.3) | |  |
| Low stroke risk | | 150 (2.0) | 38 (2.0) | 188 (2.0) | | 0.989 |
| Digoxin dosage (µg) – *mean (SD*^b^*)* | | 65.6 (24.2) | 65.8 (24.6) | 65.7 (24.3) | | 0.811 |

^a^CKD = chronic kidney disease. ^b^N **=** number. ^c^SD= standard deviation. ^d^ref. *=* reference. ^e^COPD = Chronic Obstructive Pulmonary Disease. ^f^RASi = Renin Angiotensin System inhibitor. ^g^NSAID = Non-Steroidal Anti- inflammatory Drugs. ^h^CHA2DS2-VASc score (C = Congestive heart failure; H = Hypertension; A = Age; D = Diabetes; S = Stroke; V = Vascular disease; sc = Sex category).
