# Supplementary material for: Effect of Chronic Kidney Diseases on Mortality among Digoxin Users Treated for Non-Valvular Atrial Fibrillation: A Nationwide Register-Based Retrospective Cohort Study
Source: PLoS One. 2016 Jul 28;11(7):e0160337. doi: 10.1371/journal.pone.0160337 (PMC4965154; doi:10.1371/journal.pone.0160337)
Supplement: S5 Table — (DOCX) [file pone.0160337.s005.docx]

**S5 Table. Second sensitivity analysis propensity score matched subpopulation (N=2740).**

| **Variable N**^b^ **(%) or Mean (SD**^c^**)** | **eGFR**^a^**≥30  (N**^b^**=2192)** | | **eGFR**^a^**<30  (N**^b^**=548)** | **Total  (N**^b^**=2740)** | **p-value** | |
| --- | --- | --- | --- | --- | --- | --- |
| Age in years – *mean (SD*^c^*)* | | 83.9 (8.5) | 84.6 (8.1) | 84.0 (8.4) | | 0.080 |
| Sex (*ref^d^. male*) | | 1390 (63.4) | 422 (77.0) | 1812 (66.1) | | <0.001 |
| Year of inclusion | |  |  |  | |  |
| 1997 to 2000 | | 425 (19.4) | 115 (21.0) | 540 (19.7) | |  |
| 2001 to 2004 | | 770 (35.1) | 191 (34.9) | 961 (35.1) | |  |
| 2005 to 2008 | | 592 (27.0) | 157 (28.6) | 749 (27.3) | |  |
| 2009 to 2012 | | 405 (18.5) | 85 (15.5) | 490 (17.9) | | 0.367 |
| Alcohol abuse | | 87 (4.0) | 15 (2.7) | 102 (3.7) | | 0.216 |
| Acute myocardial infarction | | 328 (15.0) | 101 (18.4) | 429 (15.7) | | 0.053 |
| Diabetes mellitus | | 432 (19.7) | 130 (23.7) | 562 (20.5) | | 0.043 |
| Arterial thrombosis | | 1493 (68.1) | 371 (67.7) | 1864 (68.0) | | 0.894 |
| Pulmonary thrombosis | | 53 (2.4) | 13 (2.4) | 66 (2.4) | | 1.000 |
| Heart failure | | 1102 (50.3) | 320 (58.4) | 1422 (51.9) | | <0.001 |
| Hypertension | | 1077 (49.1) | 235 (42.9) | 1312 (47.9) | | 0.010 |
| COPD^e^ | | 506 (23.1) | 124 (22.6) | 630 (23.0) | | 0.865 |
| Liver disease | | 53 (2.4) | 11 (2.0) | 64 (2.3) | | 0.681 |
| Peripheral arterial disease | | 227 (10.4) | 70 (12.8) | 297 (10.8) | | 0.121 |
| Stroke | | 1598 (72.9) | 398 (72.6) | 1996 (72.8) | | 0.940 |
| Syncope | | 186 (8.5) | 55 (10.0) | 241 (8.8) | | 0.288 |
| Ventricular Arrhythmias | | 23 (1.0) | 7 (1.3) | 30 (1.1) | | 0.818 |
| Lipid modifying agents | | 193 (8.8) | 54 (9.9) | 247 (9.0) | | 0.494 |
| Loop diuretic | | 1361 (62.1) | 390 (71.2) | 1751 (63.9) | | < 0.001 |
| RASi^f^ | | 1521 (69.4) | 353 (64.4) | 1874 (68.4) | | 0.029 |
| Low dose aspirin | | 1301 (59.4) | 313 (57.1) | 1614 (58.9) | | 0.367 |
| Warfarin | | 22 (1.0) | 7 (1.3) | 29 (1.1) | | 0.744 |
| Diabetes mellitus medication | | 358 (16.3) | 104 (19.0) | 462 (16.9) | | 0.157 |
| Antithrombotic therapy | | 896 (40.9) | 219 (40.0) | 1115 (40.7) | | 0.734 |
| COPD^e^ drugs | | 286 (13.0) | 64 (11.7) | 350 (12.8) | | 0.431 |
| NSAIDs^g^ | | 384 (17.5) | 101 (18.4) | 485 (17.7) | | 0.661 |
| CHA2DS2VASc^h^ – *mean (SD*^c^*)* | | 5.6 (2.1) | 5.8 (1.9) | 5.6 (2.1) | | 0.078 |
| Stroke risk (CHA2DS2-VASc^h^ score) | |  |  |  | |  |
| High stroke risk | | 2052 (93.6) | 523 (95.4) | 2575 (94.0) | |  |
| Medium stroke risk | | 96 (4.4) | 16 (2.9) | 112 (4.1) | |  |
| Low stroke risk | | 44 (2.0) | 9 (1.6) | 53 (1.9) | | 0.254 |
| Digoxin dosage (µg) – *mean (SD*^c^*)* | | 65.6 (24.2) | 65.8 (24.6) | 65.7 (24.3) | | 0.811 |

^a^eGFR = estimated Glomerular Filtration Rate. ^b^N **=** number. ^c^SD= standard deviation. ^d^ref. *=* reference. ^e^COPD = Chronic Obstructive Pulmonary Disease. ^f^RASi = Renin Angiotensin System inhibitor. ^g^NSAID = Non-Steroidal Anti-inflammatory Drugs. ^h^CHA2DS2-VASc score (C = Congestive heart failure; H = Hypertension; A = Age; D = Diabetes; S = Stroke; V = Vascular disease; sc = Sex category).
