# Supplementary material for: Effect of Chronic Kidney Diseases on Mortality among Digoxin Users Treated for Non-Valvular Atrial Fibrillation: A Nationwide Register-Based Retrospective Cohort Study
Source: PLoS One. 2016 Jul 28;11(7):e0160337. doi: 10.1371/journal.pone.0160337 (PMC4965154; doi:10.1371/journal.pone.0160337)
Supplement: S7 Table — (DOCX) [file pone.0160337.s007.docx]

**S7 Table. Third sensitivity analysis – part 2 propensity score matched subpopulation (N=8195).**

| **Variable N**^b^**(%) or Mean (SD**^c^**)** | **No CKD**^a^ **(N**^b^**=6556)** | | **CKD**^a^ **(N**^b^**=1639)** | **Total  (N**^b^**=8195)** | **p-value** | |
| --- | --- | --- | --- | --- | --- | --- |
| Age in years – *mean (SD*^c^*)* | | 81.3 (9.9) | 81.1 (9.7) | 81.3 (9.8) | | 0.382 |
| Sex (*ref^d^. male*) | | 3833 (58.5) | 984 (60.0) | 4817 (58.8) | | 0.259 |
| Year of inclusion | |  |  |  | |  |
| 1997 to 2000 | | 2164 (33.0) | 521 (31.8) | 2685 (32.8) | |  |
| 2001 to 2004 | | 2036 (31.1) | 513 (31.3) | 2549 (31.1) | |  |
| 2005 to 2008 | | 1785 (27.2) | 458 (27.9) | 2243 (27.4) | |  |
| 2009 to 2012 | | 571 (8.7) | 147 (9.0) | 718 (8.8) | | 0.809 |
| Alcohol abuse | | 353 (5.4) | 95 (5.8) | 448 (5.5) | | 0.552 |
| Acute myocardial infarction | | 1044 (15.9) | 263 (16.0) | 1307 (15.9) | | 0.934 |
| Diabetes mellitus | | 2321 (35.4) | 592 (36.1) | 2913 (35.5) | | 0.608 |
| Arterial thrombosis | | 2019 (30.8) | 500 (30.5) | 2519 (30.7) | | 0.843 |
| Pulmonary thrombosis | | 199 (3.0) | 49 (3.0) | 248 (3.0) | | 0.987 |
| Heart failure | | 3767 (57.5) | 948 (57.8) | 4715 (57.5) | | 0.801 |
| Hypertension | | 3721 (56.8) | 945 (57.7) | 4666 (56.9) | | 0.528 |
| COPD^e^ | | 1690 (25.8) | 432 (26.4) | 2122 (25.9) | | 0.654 |
| Liver disease | | 261 (4.0) | 69 (4.2) | 330 (4.0) | | 0.725 |
| Peripheral arterial disease | | 951 (14.5) | 256 (15.6) | 1207 (14.7) | | 0.272 |
| Stroke | | 1748 (26.7) | 432 (26.4) | 2180 (26.6) | | 0.826 |
| Syncope | | 547 (8.3) | 137 (8.4) | 684 (8.3) | | 1.000 |
| Ventricular Arrhythmias | | 48 (0.7) | 12 (0.7) | 60 (0.7) | | 1.000 |
| Lipid modifying agents | | 474 (7.2) | 126 (7.7) | 600 (7.3) | | 0.559 |
| Loop diuretic | | 4481 (68.3) | 1126 (68.7) | 5607 (68.4) | | 0.808 |
| RASi^f^ | | 1960 (29.9) | 488 (29.8) | 2448 (29.9) | | 0.947 |
| Low dose aspirin | | 2617 (39.9) | 655 (40.0) | 3272 (39.9) | | 0.995 |
| Warfarin | | 112 (1.7) | 26 (1.6) | 138 (1.7) | | 0.813 |
| Diabetes mellitus medication | | 1666 (25.4) | 428 (26.1) | 2094 (25.6) | | 0.582 |
| Antithrombotic therapy | | 3589 (54.7) | 893 (54.5) | 4482 (54.7) | | 0.872 |
| COPD^e^ drugs | | 844 (12.9) | 211 (12.9) | 1055 (12.9) | | 1.000 |
| NSAIDs^g^ | | 903 (13.8) | 237 (14.5) | 1140 (13.9) | | 0.498 |
| CHA2DS2VASc^h^– *mean (SD*^c^*)* | | 5.7 (2.3) | 5.6 (2.3) | 5.7 (2.3) | | 0.509 |
| Stroke risk (CHA2DS2-VASc^h^ score) | |  |  |  | |  |
| High stroke risk | | 6161 (94.0) | 1538 (93.8) | 7699 (93.9) | |  |
| Medium stroke risk | | 282 (4.3) | 69 (4.2) | 351 (4.3) | |  |
| Low stroke risk | | 113 (1.7) | 32 (2.0) | 145 (1.8) | | 0.812 |
| Digoxin dosage (µg) – *mean (SD*^c^*)* | | 65.6 (24.0) | 65.4 (23.0) | 65.6 (23.8) | | 0.712 |

^a^CKD = chronic kidney disease. ^b^N=number. ^c^SD= standard deviation. ^d^ref. *=* reference. ^e^COPD = Chronic Obstructive Pulmonary Disease. ^f^RASi = Renin Angiotensin System inhibitor. ^g^NSAID = Non-Steroidal Anti-inflammatory Drugs. ^h^CHA2DS2-VASc score (C = Congestive heart failure; H = Hypertension; A = Age; D = Diabetes; S = Stroke; V = Vascular disease; sc = Sex category).
