# Supplementary material for: Effect of Chronic Kidney Diseases on Mortality among Digoxin Users Treated for Non-Valvular Atrial Fibrillation: A Nationwide Register-Based Retrospective Cohort Study
Source: PLoS One. 2016 Jul 28;11(7):e0160337. doi: 10.1371/journal.pone.0160337 (PMC4965154; doi:10.1371/journal.pone.0160337)
Supplement: S9 Table — (DOCX) [file pone.0160337.s009.docx]

**S9 Table. Third sensitivity analysis – part 3 propensity score matched subpopulation (N=2530).**

| **Variable N^b^(%) or Mean (SD**^c^**)** | **eGFR**^a^**≥30  (N^b^=2024)** | | **eGFR**^a^**<30  (N^b^=506)** | **Total  (N^b^=2530)** | **p-value** | |
| --- | --- | --- | --- | --- | --- | --- |
| Age in years – *mean (SD*^c^*)* | | 83.7 (8.6) | 84.5 (8.0) | 83.9 (8.5) | | 0.060 |
| Sex (*ref^d^. male*) | | 1281 (63.3) | 390 (77.1) | 1671 (66.0) | | <0.001 |
| Year of inclusion | |  |  |  | |  |
| 1997 to 2000 | | 418 (20.7) | 115 (22.7) | 533 (21.1) | |  |
| 2001 to 2004 | | 779 (38.5) | 191 (37.7) | 970 (38.3) | |  |
| 2005 to 2008 | | 613 (30.3) | 157 (31.0) | 770 (30.4) | |  |
| 2009 to 2012 | | 214 (10.6) | 43 (8.5) | 257 (10.2) | | 0.444 |
| Alcohol abuse | | 69 (3.4) | 12 (2.4) | 81 (3.2) | | 0.296 |
| Acute myocardial infarction | | 311 (15.4) | 97 (19.2) | 408 (16.1) | | 0.044 |
| Diabetes mellitus | | 403 (19.9) | 124 (24.5) | 527 (20.8) | | 0.026 |
| Arterial thrombosis | | 649 (32.1) | 163 (32.2) | 812 (32.1) | | 0.991 |
| Pulmonary thrombosis | | 50 (2.5) | 13 (2.6) | 63 (2.5) | | 1.000 |
| Heart failure | | 1035 (51.1) | 302 (59.7) | 1337 (52.8) | | <0.001 |
| Hypertension | | 990 (48.9) | 284 (56.1) | 1274 (50.4) | | 0.004 |
| COPD^e^ | | 454 (22.4) | 111 (21.9) | 565 (22.3) | | 0.857 |
| Liver disease | | 48 (2.4) | 10 (2.0) | 58 (2.3) | | 0.714 |
| Peripheral arterial disease | | 211 (10.4) | 66 (13.0) | 277 (10.9) | | 0.107 |
| Stroke | | 550 (27.2) | 137 (27.1) | 687 (27.2) | | 1.000 |
| Syncope | | 168 (8.3) | 54 (10.7) | 222 (8.8) | | 0.109 |
| Ventricular Arrhythmias | | 21 (1.0) | 7 (1.4) | 28 (1.1) | | 0.668 |
| Lipid modifying agents | | 156 (7.7) | 44 (8.7) | 200 (7.9) | | 0.519 |
| Loop diuretic | | 1237 (61.1) | 361 (71.3) | 1598 (63.2) | | <0.001 |
| RASi^f^ | | 608 (30.0) | 183 (36.2) | 791 (31.3) | | 0.009 |
| Low dose aspirin | | 850 (42.0) | 223 (44.1) | 1073 (42.4) | | 0.426 |
| Warfarin | | 24 (1.2) | 7 (1.4) | 31 (1.2) | | 0.892 |
| Diabetes mellitus medication | | 323 (16.0) | 99 (19.6) | 422 (16.7) | | 0.060 |
| Antithrombotic therapy | | 1187 (58.6) | 302 (59.7) | 1489 (58.9) | | 0.708 |
| COPD^e^ drugs | | 257 (12.7) | 55 (10.9) | 312 (12.3) | | 0.296 |
| NSAIDs^g^ | | 351 (17.3) | 95 (18.8) | 446 (17.6) | | 0.489 |
| CHA2DS2VASc^h^– *mean (SD*^c^*)* | | 5.6 (2.2) | 5.8 (1.9) | 5.6 (2.1) | | 0.037 |
| Stroke risk (CHA2DS2-VASc^h^score) | |  |  |  | |  |
| High stroke risk | | 1893 (93.5) | 485 (95.8) | 2378 (94.0) | |  |
| Medium stroke risk | | 85 (4.2) | 12 (2.4) | 97 (3.8) | |  |
| Low stroke risk | | 46 (2.3) | 9 (1.8) | 55 (2.2) | | 0.121 |
| Digoxin dosage (µg) – *mean (SD*^c^*)* | | 65.6 (24.0) | 65.4 (23.0) | 65.6 (23.8) | | 0.712 |

^a^eGFR = estimated Glomerular Filtration Rate. ^b^N=number. ^c^SD= standard deviation. ^d^ref. *=* reference. ^e^COPD = Chronic Obstructive Pulmonary Disease. ^f^RASi = Renin Angiotensin System inhibitor. ^g^NSAID = Non-Steroidal Anti- inflammatory Drugs. ^h^CHA2DS2-VASc score (C = Congestive heart failure; H = Hypertension; A = Age; D = Diabetes; S = Stroke; V = Vascular disease; sc = Sex category).
