# Supplementary material for: Effect of Chronic Kidney Diseases on Mortality among Digoxin Users Treated for Non-Valvular Atrial Fibrillation: A Nationwide Register-Based Retrospective Cohort Study
Source: PLoS One. 2016 Jul 28;11(7):e0160337. doi: 10.1371/journal.pone.0160337 (PMC4965154; doi:10.1371/journal.pone.0160337)
Supplement: S10 Table — (DOC) [file pone.0160337.s010.doc]

**S10 Table. Fifth sensitivity analysis population (N=37,981)**.

| **Variable N^b^(%) or Mean (SD**^c^**)** | **No CKD**^a^ **(N^b^=37,256)** | | **CKD**^a^ **(N^b^=725)** | **Total  (N^b^=37,981)** | **p-value** | |
| --- | --- | --- | --- | --- | --- | --- |
| Age in years – *mean (SD*^c^*)* | | 81.7 (10.5) | 81.0 (9.7) | 81.7 (10.5) | | 0.086 |
| Sex (*ref^d^. male*) | | 17906 (48.1) | 469 (64.7) | 18375 (48.4) | | <0.001 |
| Year of inclusion | |  |  |  | |  |
| 1997 to 2000 | | 12659 (34.0) | 167 (23.0) | 12826 (33.8) | |  |
| 2001 to 2004 | | 10308 (27.7) | 191 (26.3) | 10499 (27.6) | |  |
| 2005 to 2008 | | 7088 (19.0) | 183 (25.2) | 7271 (19.1) | |  |
| 2009 to 2012 | | 7201 (19.3) | 184 (25.4) | 7385 (19.4) | | <0.001 |
| Alcohol abuse | | 1778 (4.8) | 42 (5.8) | 1820 (4.8) | | 0.235 |
| Acute myocardial infarction | | 3913 (10.5) | 136 (18.8) | 4049 (10.7) | | <0.001 |
| Diabetes mellitus | | 5120 (13.7) | 229 (31.6) | 5349 (14.1) | | <0.001 |
| Arterial thrombosis | | 10617 (28.5) | 203 (28.0) | 10820 (28.5) | | 0.800 |
| Pulmonary thrombosis | | 684 (1.8) | 21 (2.9) | 705 (1.9) | | 0.050 |
| Heart failure | | 13673 (36.7) | 445 (61.4) | 14118 (37.2) | | <0.001 |
| Hypertension | | 13545 (36.4) | 468 (64.6) | 14013 (36.9) | | <0.001 |
| COPD^e^ | | 7533 (20.2) | 214 (29.5) | 7747 (20.4) | | <0.001 |
| Liver disease | | 882 (2.4) | 36 (5.0) | 918 (2.4) | | <0.001 |
| Peripheral arterial disease | | 3020 (8.1) | 113 (15.6) | 3133 (8.2) | | <0.001 |
| Stroke | | 9343 (25.1) | 174 (24.0) | 9517 (25.1) | | <0.001 |
| Syncope | | 2614 (7.0) | 61 (8.4) | 2675 (7.0) | | 0.166 |
| Ventricular Arrhythmias | | 248 (0.7) | 8 (1.1) | 256 (0.7) | | 0.231 |
| Lipid modifying agents | | 2207 (5.9) | 80 (11.0) | 2287 (6.0) | | <0.001 |
| Loop diuretic | | 17248 (46.3) | 514 (70.9) | 17762 (46.8) | | <0.001 |
| RASi^f^ | | 7986 (21.4) | 213 (29.4) | 8199 (21.6) | | <0.001 |
| Low dose aspirin | | 13547 (36.4) | 267 (36.8) | 13814 (36.4) | | 0.826 |
| Warfarin | | 731 (2.0) | 9 (1.2) | 740 (1.9) | | 0.209 |
| Diabetes mellitus medication | | 4242 (11.4) | 132 (18.2) | 4374 (11.5) | | <0.001 |
| Antithrombotic therapy | | 19614 (52.6) | 400 (55.2) | 20014 (52.7) | | 0.189 |
| COPD^e^ drugs | | 5035 (13.5) | 104 (14.3) | 5139 (13.5) | | 0.553 |
| NSAIDs^g^ | | 5111 (13.7) | 88 (12.1) | 5199 (13.7) | | 0.241 |
| CHA2DS2VASc^h^ – *mean (SD*^c^*)* | | 5.0 (2.4) | 5.5 (2.2) | 5.1 (2.4) | | 0.037 |
| Stroke risk (CHA2DS2-VASc^h^ score) | |  |  |  | |  |
| High stroke risk | | 32270 (86.6) | 677 (93.4) | 32947 (86.7) | |  |
| Medium stroke risk | | 2561 (6.9) | 34 (4.7) | 2595 (6.8) | |  |
| Low stroke risk | | 2425 (6.5) | 14 (1.9) | 2439 (6.4) | | <0.001 |
| Digoxin dosage (µg) – *mean (SD*^c^*)* | | 73.5 (44.1) | 64.1 (17.0) | 73.3 (43.8) | | <0.001 |

^a^CKD = chronic kidney disease. ^b^N=number. ^c^SD= standard deviation. ^d^ref. *=* reference. ^e^COPD = Chronic Obstructive Pulmonary Disease. ^f^RASi = Renin Angiotensin System inhibitor. ^g^NSAID = Non-Steroidal Anti-inflammatory Drugs. ^h^CHA2DS2-VASc score (C = Congestive heart failure; H = Hypertension; A = Age; D = Diabetes; S = Stroke; V = Vascular disease; sc = Sex category).
